# 617 Indocyanine Green: Harnessing Novel Methods to Identify Burn Wound Healing Potential

**DOI:** 10.1093/jbcr/irac012.245

**Published:** 2022-03-23

**Authors:** Jocelyn C Zajac, Angela Gibson, Aiping Liu, Sameeha E Hassan

**Affiliations:** University of Wisconsin School of Medicine and Public Health, Madison, Wisconsin; University of Wisconsin School of Medicine and Public Health, Madison, Wisconsin; University of Wisconsin-Madison, Madison, Wisconsin; University of Wisconsin School of Medicine and Public Health, Madison, Wisconsin

## Abstract

**Introduction:**

Objective determination of burn wound healing potential remains elusive and significantly impacts decision making for surgery, the extent of tissue excised intraoperatively and the use of donor site-sparing alternative tissue therapies. Indocyanine green angiography (ICGA) has promise as an adjunct to evaluate healing potential, but feasibility has limited adoption in clinical practice. Delayed fluorescence imaging of indocyanine green (ICG), in a method called second-window ICG (SWIG), is a new technique used intraoperatively to guide tumor resection via increased peritumoral endothelial permeability. The objective of this study is to examine ICGA and SWIG fluorescence in burns requiring excision and grafting, and to correlate SWIG fluorescence to microscopic localization of inflamed and necrotic tissue.

**Methods:**

Deep partial thickness, indeterminate depth or full thickness burns were identified in adult patients scheduled for excision and grafting. 24 hours prior to surgery, baseline bright light and fluorescence images were obtained before the administration of up to 5 mg/kg ICG intravenously. ICGA was performed within 5 minutes of infusion initiation. On the day of surgery, bright light and SWIG fluorescence images were obtained before and after burn excision. The excised tissue was imaged *ex-vivo* to determine the presence of fluorescence in the tissue compared to that remaining within the wound bed. Excised tissue was processed for histologic analysis of cellular architecture, viability, inflammation and necrosis. Macroscopic ICGA and SWIG fluorescence images were compared to the associated microscopic tissue sections to determine the presence of inflammatory infiltrate, localization of non-viable tissue, and co-localization of ICG fluorescence.

**Results:**

ICGA imaging performed preoperatively demonstrated variable fluorescence throughout the burns without a clear cutoff value to delineate deep partial versus full thickness burns. SWIG imaging revealed a speckled fluorescence pattern prior to burn excision that became diffuse after excision suggesting a potential utility of SWIG to intraoperatively identify excision completion. ICGA and SWIG fluorescence demonstrated an inverse relationship, and SWIG fluorescence was associated with non-viable tissue.

**Conclusions:**

ICGA imaging alone was unreliable to delineate the need for surgical intervention. SWIG imaging of burn injuries may represent a valuable tool to guide the extent of excision intraoperatively and reduce unnecessary excision of viable tissue. Further studies are needed to understand SWIG fluorescence at the inflammation-necrosis border and how ICGA fluorescence along with SWIG can synergistically improve detection of healing potential in burn patients.

